# Low-dose cyclooxygenase-2 (COX-2) inhibitor celecoxib plays a protective role in the rat model of neonatal necrotizing enterocolitis

**DOI:** 10.1080/21655979.2021.1980646

**Published:** 2021-09-21

**Authors:** Ling Sun

**Affiliations:** Neonatal Intensive Care Unit, Yantaishan Hospital, Yantai, China

**Keywords:** Necrotizing enterocolitis, cox-2, celecoxib

## Abstract

This study aims to investigate the effects of the cyclooxygenase-2 (COX-2) inhibitor celecoxib on neonatal necrotizing enterocolitis (NEC) in rats. After treatment with a low dose of celecoxib (0.5, 1, or 1.5 mg/kg), pathological changes in the ileum and the levels of oxidative stress and inflammatory factors in NEC rats were compared. Enzyme-linked immunosorbent assay (ELISA) was employed to detect inflammatory factors, terminal deoxyribonucleotidyl transferase (TdT)-mediated biotin-16-dUTP nick-end labeling (TUNEL) staining was employed to assess apoptotic epithelial cells in the ileum, and real-time quantitative polymerase chain reaction (qRT-PCR) and Western blotting were used to quantify gene and protein expression, respectively. The incidences of NEC rats in the 0.5, 1 and 1.5 mg/kg celecoxib groups were lower than in the model group (100%). Celecoxib improved the histopathology of the ileum in NEC rats. Moreover, low doses of celecoxib relieved oxidative stress and inflammation in NEC rats, as evidenced by decreased tumor necrosis factor-α (TNF-α), interferon-γ (IFN-γ), total oxidation state (TOS), malondialdehyde (MDA) and oxidative stress index (OSI), as well as increased interleukin-10 (IL-10), total antioxidant status (TAS), superoxide dismutase (SOD) and glutathione peroxidase (GPx). With increasing celecoxib doses (0.5, 1, or 1.5 mg/kg), the amount of apoptotic epithelial cells in the ileum of NEC rats gradually declined and Caspase-3 expression was reduced. The low dose of the COX-2 inhibitor celecoxib ameliorated the histopathologic conditions of the ileum, alleviated oxidative stress and inflammation, and reduced apoptotic epithelial cells in NEC rats, thereby making it a potential therapy for NEC.

## Introduction

Neonatal necrotizing enterocolitis (NEC) is the major gastrointestinal cause of neonatal mortality, mainly affecting premature and/or low birth-weight infants; clinical symptoms include vomiting, diarrhea and bloody stool, and pathological characteristics of ulcers, hemorrhage and necrosis of the small intestine (especially the end of the ileum) are present [1–3]. The estimated NEC incidence ranges from 0.3 to 2.4 infants per 1000 live births, accounting for approximately 7%-11% of neonates weighing < 1,500 g [1]. Unfortunately, the survival rate of NEC infants with birth weights less than 1,500 g is only approximately 70% [4]. Moreover, 25% of NEC infants develop short bowel syndrome (SBS) and neurodevelopmental disorders (NDs) [5]. Definitive NEC may require medical (abdominal decompression, bowel rest, broad-spectrum intravenous antibiotics, and intravenous hyperalimentation) or surgical (drain placement, exploratory laparotomy with resection of diseased bowel, and enterostomy with creation of a stoma) management [6]. With improvements in medical science and technology, the survival rate of premature infants continues to increase, but once surgery is required, the outcome may be poor [6]. Therefore, current research is still focused on investigating the pathogenesis of NEC in depth

Cyclooxygenase (COX) is a key enzyme in the conversion of arachidonic acid to prostaglandin (PG) H2; three subtypes exist, namely, COX-1, COX-2 and COX-3 [7]. Among these three subtypes, COX-2 is an inducible enzyme that is highly expressed in various human inflammatory diseases, such as asthma [8], osteoarthritis [9], chronic gastritis [10] and inflammogenesis of cancer [11]. Notably, a recent study demonstrated increased COX-2 expression in NEC animals with a significant loss of mucosa [12]. Similarly, the upregulated expression of COX-2 was also found in NEC pups [13]. In addition, some drugs that inhibit COX-2 expression, such as celecoxib, may play a therapeutic role in NEC [14,15]celecoxib. Celecoxib is a nonsteroidal anti-inflammatory drug (NASID) that functions as a COX-2-specific inhibitor [16,17] and can block the conversion of arachidonic acid to prostaglandins to increase anti-inflammatory and analgesic activity [18], protect gastric mucosa [19], maintain renal blood flow [20], and regulate platelet aggregation [21]. More importantly, Golden J *et al*. also revealed that a low dose celecoxib could alleviate NEC pathology [22].

We therefore hypothesized that celecoxib may be a potential useful treatment for NEC. To investigate the possible mechanism by which celecoxib affects NEC in rats, low doses of celecoxib (0.5 mg/kg, 1 mg/kg and 1.5 mg/kg) were intraperitoneally administered to neonatal NEC rats, and the anti-inflammatory, anti-oxidative stress and anti-apoptotic effects were determined.

## Materials and methods

### Ethics statement

This study was conducted in compliance with the Guide for the Care and Use of Laboratory Animals [23], and all animal experiments were performed under the supervision of the Medical Ethics Committee of Laboratory Animals in our hospital.

### Experimental animals

In this study, 100 healthy newborn Sprague–Dawley (SD) rats were used to perform animal experiments; animals were kept in an environmentally controlled room (24 ± 0.5°C and humidity 50%-55%) with a standard photoperiod (12 h light and 12 h dark). The neonatal rats were fed freely on breast milk by their mothers, and a standard rodent diet and tap water were provided for rat mothers ad libitum.

### NEC model establishment

Neonatal rats were randomly divided into five groups: normal, model, 0.5 mg/kg celecoxib, 1 mg/kg celecoxib, and 1.5 mg/kg celecoxib groups, with 20 rats in each group. All neonatal rats developed experimental NEC except for those in the normal group. NEC was induced by following the procedures described in a previous study [24]. Specifically, rats were exposed to 100% nitrogen gas for 120 s and then placed at 4°C for 10 min two times a day for three consecutive days. Approximately 30 min before NEC induction, SD rats in the celecoxib groups were treated once daily with 0.5 mg/kg, 1 mg/kg or 1.5 mg/kg celecoxib dissolved in dimethyl sulfoxide (DMSO) in normal saline via intraperitoneal (i.p.) injection [22]. Celecoxib (1,098,504, Sigma-Aldrich Trading Co. Ltd., Shanghai, China) was preserved in a cool place. Rats in the normal group were given normal saline as a control. All rats were weighed every day, and the number of deaths per day was recorded. On the 4^th^ day of the experiment, the living rats were sacrificed via decapitation.

### Enzyme-linked immuno sorbent assay (ELISA)

Blood was withdrawn from the retro-orbital plexus under light ether anesthesia for biochemical estimations of serum interleukin-10 (rat IL-10 ELISA kit, ERA23RBX5, Invitrogen, USA), tumor necrosis factor-α (rat TNF-α ELISA kit, BMS622, Invitrogen, USA) and interferon-γ (rat INF-γ ELISA kit, RAB0227, Invitrogen, USA) expression.).

### HE staining

Ileum tissue samples were fixed in 10% formalin, dehydrated with gradient alcohol, hyalinized twice with xylene, embedded in paraffin, and sliced into 4-μm-thick sections. Paraffin sections were routinely deparaffinized in water, stained in hematoxylin for 10 min at room temperature, differentiated by 1% hydrochloric ethanol for 1 min, and stained in eosin (at room temperature). Tissue sections were then immediately dehydrated using gradient alcohol, hyalinized with xylene, mounted with neutral resin, photographed and observed under an optical microscope for morphological changes. The histopathological changes in the ileum tissues were assessed by a blinded evaluator and scored (0–4) accordingly. For the quantification of NEC incidence, rats with histological scores lower than 2 were classified as having no NEC, while those with histological scores equal to or higher than 2 were classified as having NEC.

### Oxidative stress index detection

Ileum tissues were ground to homogenate in saline (1 g in 85 ml) and centrifuged at 4000 × g for 20 min to collect the upper supernatant for subsequent analysis. Total oxidation state (TOS) and total antioxidant status (TAS) was determined using the colorimetric method (Rel Assay Diagnostics kit). The oxidative stress index (OSI) was calculated according to the formula OSI = TOS/TAC. The levels of malondialdehyde (MDA), superoxide dismutase (SOD) and glutathione peroxidase (GPx) were detected by following instructions in previous studies [25–27].

### Terminal deoxynucleotidyl transferase dUTP nick end labeling (TUNEL) assay

Terminal ileum slides were stained and detected in accordance with the manufacturer’s instructions for the TUNEL assay kit (Promega, Madison, WI). A Zeiss Observer Z1 fluorescence microscope was used to capture tissue staining images at 40×, and image analysis was performed using ImageJ software (NIH). The apoptotic index was evaluated as the proportion of DAPI^+^ (blue) cells stained for TUNEL^+^ (green) per high-power field (HPF), and at least 4 HPFs were quantified for each sample.

### Real-time quantitative polymerase chain reaction (qRT–PCR)

Total RNA was extracted using a TRIzol kit, RNA concentration was quantified with an ultraviolet spectrophotometer, and RNA was reverse transcribed into cDNA with a PrimeScript RT Reagent Kit (Takara Biotechnology, Dalian, China). Primers were designed with Primer 5.0 software, which was synthesized by GenScript Co., Ltd. (Nanjing, China). The gene sequences were as follows: TNF-α: Forward (5ʹ-3ʹ): ATGTGGAACTGGCAGAGGAG, Reverse (3ʹ-5ʹ): TGGAACTGATGAGAGGGAGC; IFN-γ: Forward (5ʹ-3ʹ): GTGTCATCGAATCGCACCTG, Reverse (3ʹ-5ʹ): GGTGACAGCTGGTGAATCAC; IL-10: Forward (5ʹ-3ʹ): CGCTTGTCCTCCTTGTCAAC, Reverse (3ʹ-5ʹ): TCAATTCTGTGGCCTGCTTG; β2-microglobulin) (B2 MB2M, internal control): Forward (5ʹ-3ʹ): AGTGTACTCTCGCCATCCAC, Reverse (3ʹ-5ʹ): CGGTGGGTGTGAATTCAGTG. PCR was performed via a SYBR-Green PCR Reagent Kit (Clontech Laboratories, Mountain View, CA) and a Bio-Rad IQ5 Real-Time System (Bio-Rad laboratories, Hercules, CA, USA). The relative expression of genes was calculated using the 2^−ΔΔCt^ method.

### Western blotting

Total proteins were extracted and protein concentration was determined using a bicinchoninic acid (BCA) kit (Sigma, USA). The protein concentration and loading volume of each sample were equalized. Sodium dodecyl sulfate-polyacrylamide gel electrophoresis (SDS-PAGE) was used to separate proteins, which were transferred to polyvinylidene fluoride (PVDF) films via a semidry transfer system (Bio–Rad, USA). The PVDF film was blocked in skim milk at room temperature, washed using phosphate-buffered saline with Tween 20 (PBST) buffer, and incubated for 1 h at room temperature with anti-cleaved caspase-3 antibody (PA5-23,921,-23,921, 1/1000 dilution, Invitrogen, USA) and anti-β-actin antibody-loading control (ab8227, 1/1000 dilution, Abcam, UK). Next, the PVDF film was washed with PBST buffer 5 times for 3 min each time and incubated for 1 h at room temperature with goat anti-rabbit IgG H&L (HRP) (ab97051, Abcam, UK) (1/20,000 dilution). After that, the film was washed with PBST buffer again 5 times (3 min/wash) before the development of target proteins using horseradish peroxidase (HRP) substrate (Bio-Rad). The relative expression of target proteins was calculated as the gray value ratio of target protein to β-actin.

### Statistical methods

Statistical data were analyzed using SPSS 22.0. Data are presented as the mean ± standard deviation. Comparisons among multiple groups were tested by one-way ANOVA or post hoc Tukey’s test. *P* < 0.05 indicated a statistically significant difference.

## Results

### Celecoxib decreased the severity of NEC in neonatal rats

**Figure 1.**
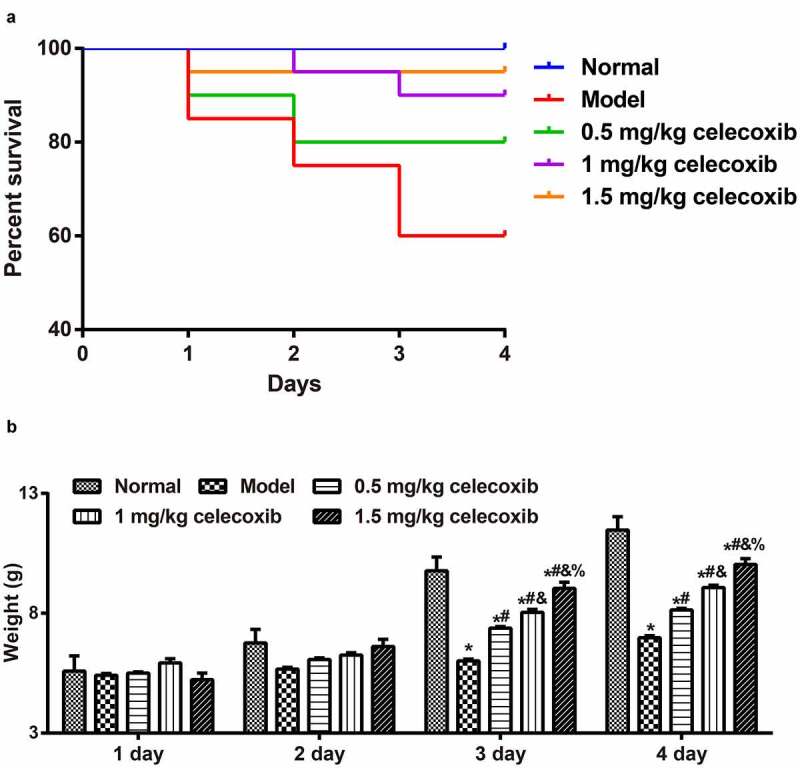
Celecoxib decreased the severity of NEC in neonatal rats

Note: A, The survival of neonatal rats in each group; B, The weight of neonatal rats in each group; *, *P* < 0.05 compared with the normal group; #, *P* < 0.05 compared with the model group; &, *P* < 0.05 compared with the 0.5 mg/kg celecoxib group; %, *P* < 0.05 compared with the 1 mg/kg celecoxib group.

To explore the protective role of celecoxibcelecoxib in neonatal NEC rats, the survival rate and the weight of rats were recorded. The number of deaths in the normal, model, 0.5 mg/kg celecoxib, 1 mg/kg celecoxib and 1.5 mg/kg celecoxib groups was 0 (0%), 8 (40%), 4 (20%), 2 (10%), and 1 (5%), respectively (Figure 1a). During NEC model establishment, the weight of neonatal rats from each group increased gradually with time; weights in the normal group increased most significantly, followed by the 1.5 mg/kg celecoxib group, 1 mg/kg celecoxib group and 0.5 mg/kg celecoxib successively, with the model group showing the lowest increase. On the 3^rd^ and 4^th^ days, there was a significant difference in the weight of neonatal rats among the groups (all *P* < 0.05, Figure 1b).)

### Celecoxib reduced NEC incidence in neonatal rats

**Figure 2.**
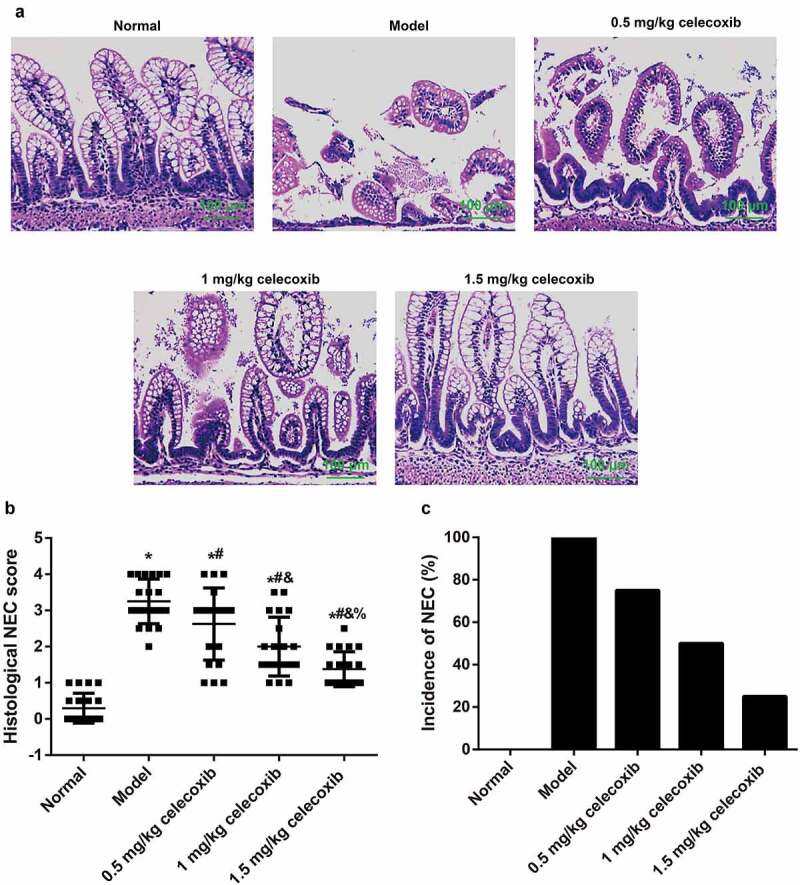
Celecoxib reduced NEC incidence in neonatal rats

Note: A, The histopathological changes of ileum tissues in neonatal rats were evaluated after HE staining; B, The ileum tissue damage was measured via an histological scoring system; C, The incidence of NEC (%) of neonatal rats in each group. *, *P* < 0.05 compared with the normal group; #, *P* < 0.05 compared with the model group; &, *P* < 0.05 compared with the 0.5 mg/kg celecoxib group; %, *P* < 0.05 compared with the 1 mg/kg celecoxib group.

The histological changes (Figure 2a), corresponding histological scores (Figure 2b), and incidence of NEC (Figure 2c) in the experimental groups were demonstrated individually and summarized to demonstrate the effect of celecoxib on NEC incidence in neonatal rats. Rats in the normal group exhibited intact intestinal villi and epithelium and normal ileum tissue structure without inflammatory cell infiltration. In contrast, rats in the model group showed necrosis, villus structure damage and transmural necrosis. After treatment with celecoxib at different dosages, NEC rats showed some improvements in histopathological changes of the ileum and exhibited with lower histopathological scores. In addition, the proportion of NEC rats with a score of 2 or greater, which is indicative of NEC, was 75% (15/20), 50% (10/20) and 25% (5/20) in the 0.5 mg/kg celecoxib, 1 mg/kg celecoxib and 1.5 mg/kg celecoxib groups, respectively; these values were significantly lower than in the model group (100%). In the normal group, the NEC incidence rate was 0% (0/20).

### Celecoxib affected inflammatory factors in the serum and ileum of NEC rats

**Figure 3.**
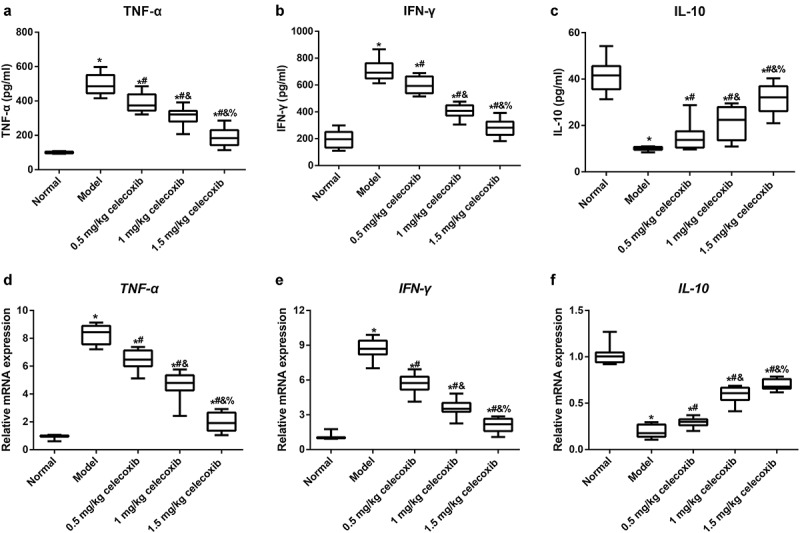
The COX-2 inhibitor celecoxib affected inflammatory factors in the serum and ileum of NEC rats

Note: A-C, The expression of TNF-α (A), INF-γ (B) and IL-10 (C) in serum of neonatal rats determined with ELISA; D-F, The mRNA expression of *TNF-α* (D), *INF-γ* (E) and *IL-10* (F) in ileum tissues of neonatal rats detected by qRT-PCR; *, *P* < 0.05 compared with the normal group; #, *P* < 0.05 compared with the model group; &, *P* < 0.05 compared with the 0.5 mg/kg celecoxib group; %, *P* < 0.05 compared with the 1 mg/kg celecoxib group.

Subsequently, to determine the anti-inflammatory role of celecoxib in neonatal NEC rats, ELISA and qRT-PCR were used to examine the expression of IL-10, TNF-α and INF-γ in the serum and ileum. The results showed that NEC rats exhibited significantly higher serum levels of TNF-α and INF-γ but lower levels of IL-10 compared with normal rats. After NEC rats were treated with 0.5 mg/kg, 1 mg/kg and 1.5 mg/kg celecoxib, the levels of TNF-α and INF-γ in serum were gradually decreased with increased IL-10 level (all *P* < 0.05, Figure 3a-c). The mRNA expression levels of the aforementioned inflammatory factors in the ileum were consistent with the ELISA results (Figure 3d-f).

### Celecoxib regulated oxidative stress in the ileum of NEC rats

**Figure 4.**
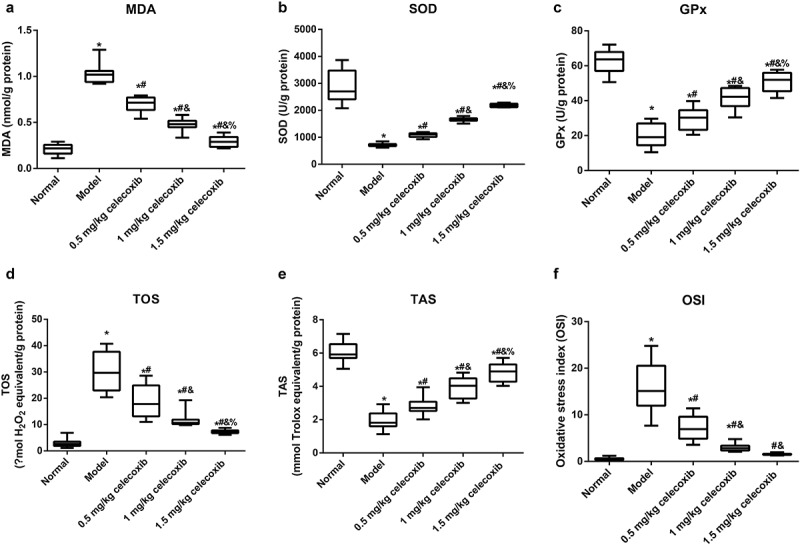
The COX-2 inhibitor celecoxib regulated the oxidative stress of ileum in NEC rats

Note: Malondialdehyde (MDA, A), Superoxide dismutase (SOD, B), Glutathione peroxidase (GPx, C), Total oxidant status (TOS, D), Total antioxidant status (TAS, E), Oxidative stress index (OSI, F), OSI = TOS/TAS; *, *P* < 0.05 compared with the normal group; #, *P* < 0.05 compared with the model group; &, *P* < 0.05 compared with the 0.5 mg/kg celecoxib group; %, *P* < 0.05 compared with the 1 mg/kg celecoxib group.

To investigate the anti-oxidative stress role of celecoxib in neonatal NEC rats, the following indicators of oxidative stress in the ileum were detected: MDA, SOD, GPx, TOS, TAS, and OSI (TOS/TAS) (Figure 4). NEC rats had increased levels of TOS and MDA and decreased TAS, SOD and GPx compared with normal rats (all *P* < 0.05). In addition, 0.5 mg/kg, 1 mg/kg and 1.5 mg/kg celecoxib alleviated oxidative stress in ileal tissues in NEC rats, accompanied by reductions in TOS and MDA and upregulation of TAS, SOD and GPx, with significant differences among treatment groups (all *P* < 0.05). Additionally, the OSI was calculated, and a higher OSI was found in the model group than in the normal group (*P* < 0.05). Compared with the model group, the OSI in neonatal rats treated with celecoxib declined dramatically (all *P* < 0.05). In comparison with the 1 mg/kg celecoxib group, OSI was higher in the 0.5 mg/kg celecoxib group and lower in the 1.5 mg/kg celecoxib group (all *P* < 0.05). However, no obvious difference was found between the 1.5 mg/kg celecoxib and normal groups in terms of the OSI (*P* > 0.05).

### Celecoxib affected the apoptosis of epithelial cells in the ileum of NEC rats

**Figure 5.**
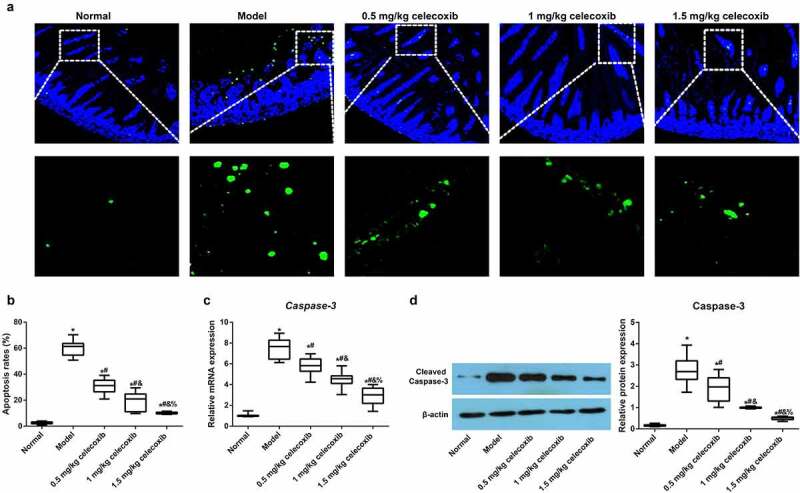
The COX-2 inhibitor celecoxib affected ileal apoptosis in NEC rats

Note: A, Immunofluorescence images of TUNEL (green fluorescence) as an indicator of apoptosis in the ileum of neonatal rats, DAPI (blue staining) served as a counterstain; B, counterstain; B, Comparison of apoptotic rates among groups (B); C, The mRNA expression of *Caspase-3* in the ileum quantified by qRT–PCR; D, The protein expression of Cleaved Caspase-3 in the ileum quantified by Western blotting; *, *P* < 0.05 compared with the normal group; #, *P* < 0.05 compared with the model group; &, *P* < 0.05 compared with the 0.5 mg/kg celecoxib group; %, *P* < 0.05 compared with the 1 mg/kg celecoxib group.

We next examined the apoptosis of epithelial cells in the ileum using TUNEL staining (Figure 5a-b), and the results demonstrated that, compared with the normal group, the model group had an increased apoptosis rate (*P* < 0.05). However, with increasing celecoxib dosage (0.5 mg/kg, 1 mg/kg and 1.5 mg/kg), the apoptosis of epithelial cells gradually decreased (all *P* < 0.05). Subsequently, the expression of *Caspase-3* and Cleaved Caspase-3 in the ileum was detected using qRT-PCR and Western blotting, respectively, and celecoxib reduced *Caspase-3* and Cleaved Caspase-3 expression in the ileum of NEC rats (all *P* < 0.05, Figure 5c-d).

## Discussion

Abdominal X-ray and ultrasound have been used to monitor the progression of NEC [28]. In general, supportive medical management alone is provided for suspected NEC (Bell stage I), while medical management is usually tried first for proven NEC (Bell stage II), and medical management and inotropic support may be required for advanced NEC (Bell stage III) [29]. Surgical treatment is considered when the patient fails to respond to medical treatment [30]. To explore out more preventative and treatment methods for NEC infants, iinvestigators have modified the protocols to establish an NEC model [31,32]. In this work, an NEC rat model was modified by adding extra hyperosmolar formula to the rat diet. The NEC incidence rate was 100%, evidenced by necrosis and loss of villi structure and transmural necrosis in the ileum of rats. These findings demonstrated that the establishment of the model was successful.

High levels of COX-2 are observed during intestinal inflammation and depend on PGE_2_-EP receptor signaling [22]. In recent years, accumulating studies have described a significant upregulation of COX-2 in NEC rats [13–15,33], indirectly suggesting that the inhibition of COX-2 could be a potential target for the treatment and prevention of NEC. As a COX-2 selective inhibitor,(0.5 mg/kg, 1 mg/kg and 1.5 mg/kg) in this study led to a weight increase in NEC model rats, reduced NEC incidence and mortality, and alleviated ileum histopathology, suggesting that a low dose of celecoxib can inhibit the development of NEC and play a protective role in NEC-related deaths, which was consistent with the finding of a previous study [22].

The inflammatory response plays an important role in NEC pathogenesis, and many inflammatory factors are involved in the progression of NEC [2,34]. In this study, a low dose of celecoxib effectively reduced TNF-α and INF-γ levels and increased IL-10 levels in the serum and ileum of NEC rats. According to a previous study, celecoxib can also reduce the levels of proinflammatory cytokines, including IL-1β, IL-6, INF-γ and TNF-α, in the cerebral cortex induced by pentylenetetrazol (PTZ) low dose of level According to a previous study, celecoxib can also reduce the level of pro-inflammatory cytokines in cerebral cortex induced by pentylenetetrazol (PTZ)[35]. Morales-Sosa M *et al*. observed the neuroprotective effect of celecoxib in neonatal SD rats and found that celecoxib can block proinflammatory proteins to reduce epilepsy susceptibility via the key HMGB1/TLR4 pathway, which is implicated participated in the evolution of diseases with inflammatory processes [36]. In addition, hyperoxia exposure increased the secretion of TNF-α and IL-6 in the bronchoalveolar lavage fluid of neonatal rats, and celecoxib significantly decreased the protein levels of these proinflammatory factors via the inhibition of the NF-κB signaling pathway [37]. The aforementioned evidence therefore demonstrates that celecoxib can inhibit the inflammatory response in NEC rats, possibly via blockade of the HMGB1-TLR4-NF-κB pathway; this will would be further investigated in the near future.

Generally, premature infants have an immature intestinal mucosal barrier and are prone to intestinal hypoxia, ischemia, and abnormal bacterial colonization, which can produce numerous oxygen free radicals and activate intestinal oxidative stress. These processes further result in the peroxidation of protein, DNA and the cell membrane, as well as excessive MDA generation, leading to tissue and cell damage [24]. The first line of defense of the body’s antioxidant system is to inhibit the formation of excessive oxygen free radicals and lipid peroxidation. Cellular, defense against oxidative stress is mediated by SOD and GPx, which can neutralize oxygen free radicals to combat oxidative stress [38]. TAS, TOS, and OSI indicate general changes in oxidative stress [39]. The upregulation of TOS and OSI and the downregulation of TAS were also observed in rat pups induced by NEC in some studies [40,41]. Recently, celecoxib was proven to inhibit epithelial hyperplasia (DNA content) and oxidative stress induced by testosterone in a rat model of prostatic hyperplasia [42]. The administration of celecoxib mitigated oxidative lipid damage, thus reducing oxidative stress to exert a therapeutic effect on renal ischemia/reperfusion injury [43]. Moreover, celecoxib administration reduced the oxidative stress-mediated risk of carcinogenesis due to an ability to boost the antioxidant defense system, as evident from the increase in SOD activity [44]. In this study, we also found that 0.5 mg/kg, 1 mg/kg and 1.5 mg/kg celecoxib mitigated oxidative stress in the ileum in NEC rats, accompanied by reductions in TOS and MDA, as well as increases in TAS, SOD and GPx, indicating that celecoxib alleviates oxidative stress to slow the development of NEC in rats. The potential mechanisms involved in the antioxidant activity of celecoxib include modulation of the inducible nitric oxide synthase pathway and a decrease in the generation of hydroxyl radicals [45].

Furthermore, obvious apoptosis of epithelial cells in the ileal tissue of NEC rats was also observed, which was consistent with the findings of a large number of domestic and foreign studies [24,34,46]. However, with increasing celecoxib dosage, apoptosis of ileal epithelial cells gradually decreased in NEC rats. As reported in a previous study, celecoxib treatment can reduce ET-18-O-CH(3)-induced breast epithelial cell death [47]. Intestinal epithelial cells treated with dimethylhydrazine hydrochloride had an increase in the number of apoptotic cells, which could be restored to normal levels by celecoxib [48]. At the molecular level, apoptosis involves the activation of select caspases that are characteristic of the signal event that initiates apoptosis [49]. Notably, there was evidence that the regulation of cellular apoptosis by celecoxib was via the mediation of caspase expression to some extent [50]. Caspase-3 (also known as CPP32, Yama or apopain), a key protease in the caspase family, is activated by various apoptosis stimulating factors. The inhibition of Caspase-3 is expected to become an important link in the treatment of diseases related to excessive cell death [51]. Similarly, the low concentration of celecoxib in our study reduced the expression of *Caspase-3* and Cleaved Caspase-3 in ileum tissues of NEC rats indicating that celecoxib can inhibit the apoptosis of epithelial cells in the ileum of NEC rats via the regulation of Caspase-3.

Our study had several limitations, including lack of an in-depth exploration of target cells in this model due to time and funding constraints. Another limitation was that the minimum effective concentration and the maximum safe concentration of celecoxib were not detected in our experiment; these should be further explored in the future.

## Conclusion

Celecoxib can reduce NEC incidence, improve histopathological changes in the ileum alleviate oxidative stress and inflammation, and reduce the apoptosis of epithelial cells in the ileum of NEC rats, and is therefore a potential therapy in the treatment of NEC.
